# Effect of Surfactant Type, Cholesterol Content and Various Downsizing Methods on the Particle Size of Niosomes

**Published:** 2018

**Authors:** Fatemeh Nowroozi, Ameneh Almasi, Jaber Javidi, Azadeh Haeri, Simin Dadashzadeh

**Affiliations:** a *Student Research Committee, School of Pharmacy, Shahid Beheshti University of Medical Sciences, Tehran, Iran. *; b *Department of Pharmaceutics and Nanotechnology, School of Pharmacy, Shahid Beheshti University of Medical Sciences, Tehran, Iran.*; c *Pharmaceutical Sciences Research Center, Shahid Beheshti University of Medical Sciences, Tehran, Iran.*

**Keywords:** Particle size, Niosomes, Probe sonication, Extrusion, High pressure homogenizer

## Abstract

The present study was conducted to investigate the performance of different size reduction techniques including probe sonication, extrusion, and high pressure homogenization for nanosizing of niosomes. Also, the effects of cholesterol content and surfactant type on the size and poly dispersity index (PDI) of the formulations were evaluated. Various niosomal formulations composed of Brij 72, Span 60, or Tween 60 were prepared and then, to reduce vesicle size and minimize the PDI, the niosomes were treated by various post-processing procedures. Surfactant type showed a significant effect on the particle size of the niosomes. The particle size of Tween 60 niosomes was significantly larger than those of Span 60 and Brij 72 niosomes (*P* < 0.05), indicating that at the same cholesterol content, niosomes composed of a surfactant with a higher HLB value show larger particle size than those with a lower HLB value. The influences of cholesterol content as well as downsizing methods, on the particle size and distribution of niosomes were significantly dependent on the surfactant composition of the niosomes. Extrusion and probe sonication were found to be the most efficient methods for size reduction of the Tween 60 and Span 60 niosomes, while for downsizing of Brij 72 niosomes, all employed methods were efficient and resulted in the approximately similar size of about 200 nm. In conclusion, the selection of an efficient method for nanosizing of niosomes and also achievement of a desired size range, and homogeneity highly depends on the niosome composition, particularly on the employed surfactant type.

## Introduction

Niosomes are spheroidal bilayers prepared from the mixtures of some non-ionic surfactants and cholesterol. Cholesterol is one of the main constituent that stabilizes the niosomes membrane (1). Nonionic surfactants are non-immunogenic, biocompatible, and biodegradable; hence, the vesicles composed of them can be used as an appropriate drug delivery system (2). Liposomes and noisomes have a similar structure composed of uni- or multilamellar spheroidal structures and both have been extensively studied; however, niosomes offer several advantages versus liposomes such as higher stability, lower cost, and facile handling and storage (3). In last decade niosomes based formulation have more attention in various nanomedicine field such as drug delivery (4), tumor treatment (5) , *etc.* (6). Particle size is one of the key features of nanoparticles that influence their distribution, clearance, circulation time, and thus their capacity for targeting to target organ (7). Kidney as a clearance system of the body can filter molecules less than 5.5 nm in size (8), the spleen is a large, highly vascular lymphoid organ that can filter larger particles of approximately 200–250 nm (9). Studies have also demonstrated that the hepatobiliary system of liver can eliminate nanoparticles that cannot be cleared by the renal system (6-100 nm) (10). The capillary vessel boundaries in the lung may arrest some nanoparticles, especially those larger than 1000 nm (9). Therefore, achieving a desired particle size with a low PDI is a common key property that should be considered in design of an effective nanocarrier. The final particle size achieved by various technologies is important. Niosomes are spherical in shape and, in general, in spite of the preparation method used, their initial size immediately after preparation is within the micro-size range. Hence, to achieve an optimum size and PDI in accordance with the clinical goal, additional size reducing processes is required (11).

Different size reducing methods, such as sonication, extrusion, and homogenization have been used in the literature to produce particles with various sizes (11). However, the effect of these methods and the relevant process parameters on the properties of niosomes has not been systemically studied yet. Sonication as either probe (12) or bath type (13), has been used as an effective method for reducing the particle size of vesicles down to a small scale. Another potential reported method for the particle size reduction is high-pressure homogenization, which has been usually at pressures above 5000 psi. This method can be used for continuous production of nanoparticles at a large scale (14-17). Extrusion is another commonly used technique particularly for liposomes, in which the suspension is passed through a set of various pore sized membrane filters (18-20). To the best of our knowledge, the effect of these methods and the relevant process parameters on the properties of niosomes has not been systemically studied yet. Therefore, the present study was conducted to investigate and compare the performance of the abovementioned different size reduction techniques for nanosizing of niosomes. In addition, the effects of cholesterol amount and the hydrophobic-lipophilic balance (HLB) and type of surfactants, as the main components of niosomes, on the particle size and PDI of the prepared vesicles as well as on the efficiency of different nanosizing techniques were examined.

## Experimental

*Materials*

Span 60, Tween 60, Brij72, cholesterol (Chol), chloroform, methanol, and isopropanol were obtained from Merck (Germany). Chemical structures, HLB values, molecular weights, and phase transition temperatures of the non-ionic surfactants used in this study are summarized in Table 1.

*Preparation of niosomes*

Niosomes were prepared with various mixtures of nonionic surfactant/cholesterol at ratios of 60:40 and 80:20, using thin film-hydration method. The compositions of the prepared niosomes were shown in Table 2. Briefly, accurately weighed quantities of the surfactant (Tween 60, Span 60 or Brij72) and cholesterol were dissolved in 8 mL of chloroform: methanol mixture (3:1, v/v) in a round-bottom flask. The organic solvents were removed under vacuum in a rotary evaporator at 60 °C for 60 min to form a thin film on the wall of the flask. Thereafter, the thin film was kept in a desiccator under vacuum for 1 h to ensure total removal of the trace solvents. The dried thin film was hydrated with 10 mL of phosphate buffered saline (PBS) either with conducting simultaneous bath sonication (405, Hwashin Liarre, Korea) for 1 h (3 cycles of 18 min) or without sonication process.

*Nano-sizing of niosomes*

**Figure 1:**
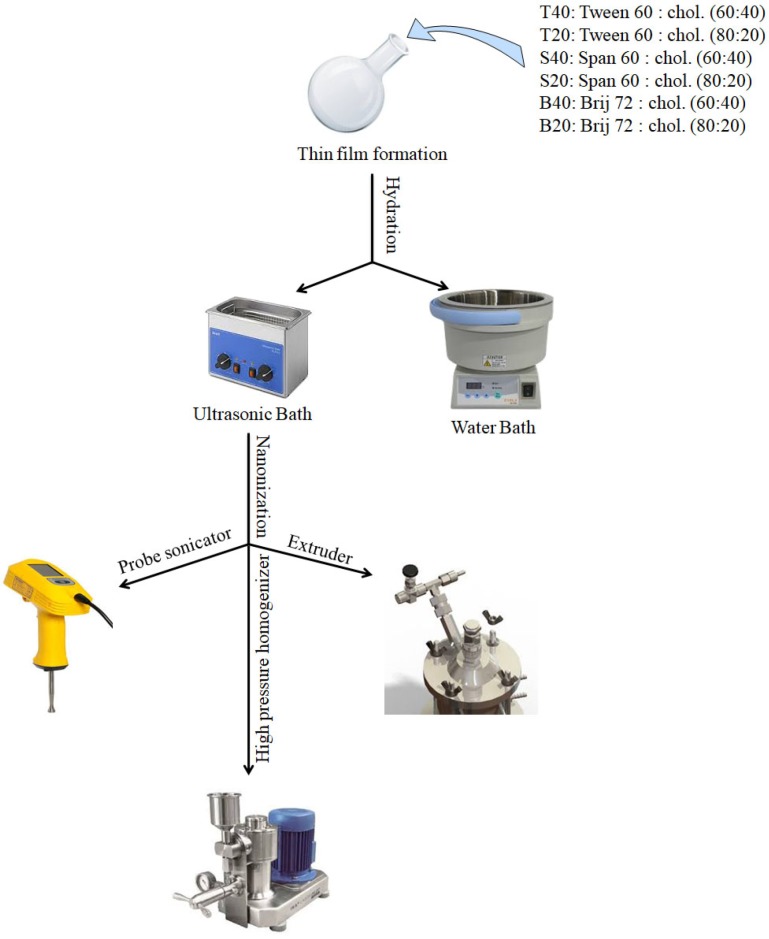
Schematic illustration of the preparation and downsizing process of niosomes

The prepared vesicles were down sized using different methods: probe sonication, high pressure homogenization, or extrusion. Schematic illustration of the preparation and down- sizing process of niosomes was shown in Figure 1.

*Probe Sonication*

Sonication of the niosome suspensions was carried out by a probe sonicator (series UP200Ht, Hielscher, Germany) with titanium probe of 1 mm diameter. The sample (3 mL) was sonicated at a power of 150 W for 15 min. In order to avoid excess heating of the sample, the beaker was immersed in an ice bath. 

*High pressure homogenization (HPH)*

Fine vesicles were formed by passing the suspensions through a high pressure homogenizer (IKA HPH 2000/4, Germany). The sample (6 mL) was fed into the HPH and was passed through the homogenization unit for 15 min at homogenization pressures ranging from 800-1000 bars. 

*Extrusion*

To obtain vesicles with smaller diameters niosomes (5 mL) were extruded 5 times through polycarbonate membranes (pore sizes of 400, 200 nm) using an extruder (Lipex 10 mL, Northern Lipid Inc., Canada).

*Particle size analysis*

The particle size and PDI of niosomes were determined by dynamic light scattering (Zetasizer Nano ZS, Malvern Instruments, England) dilution with PBS (pH 7.4). All experiments were repeated three-time. Standard deviation of the average was used to estimate the repeatability of the measurements.

**Table 1:** Chemical structures, HLB values, molecular weights and phase transition temperatures of the non-ionic surfactants used in this study (5).

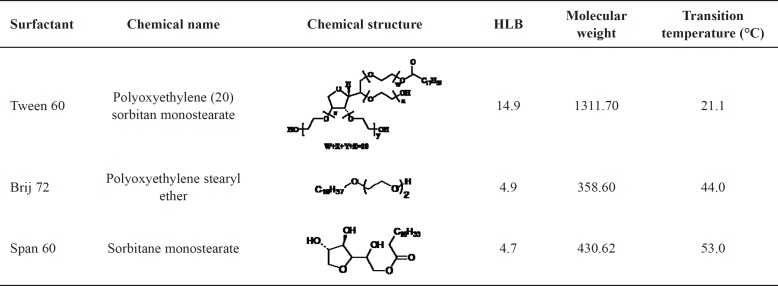

**Table 2:** Composition of the prepared niosomes

**Formulation**	**Niosome composition(molar ratio)**
T-40	Tween 60: Chol (60:40)
T-20	Tween 60: Chol (80:20)
S-40	Span 60: Chol (60:40)
S-20	Span 60: Chol (80:20)
B-40	Brij 72: Chol (60:40)
B-20	Brij 72: Chol (80:20)

**Table 3:** Particle size and PDI of niosomal formulations prepared by thin film hydration in either the presence or absence of the bath sonication treatment (Mean ± SD, n = 3).

**Formulation**	**With bath sonication**		**Without bath sonication**		***P*** **-value**	
	**Size**	**PDI**	**Size**	**PDI**	**Size**	**PDI**
T40	646 ± 4.54	0.553 ± 0.004	641 ± 6.98	0.766 ± 0.006	*P *> 0.05	*P *< 0.01
T20	601 ± 5.65	0.212 ± 0.007	615 ± 2.56	0.512 ± 0.004	*P *> 0.05	*P *< 0.001
S40	426 ± 2.18	0.363 ± 0.003	433 ± 4.50	0.415 ± 0.002	*P *> 0.05	*P *< 0.05
S20	591 ± 5.08	0.410 ± 0.004	595 ± 7.12	0.598 ± 0.005	*P *> 0.05	*P *< 0.05
B40	466 ± 7.32	0.347 ± 0.005	459 ± 4.21	0.556 ± 0.004	*P *> 0.05	*P *< 0.01
B20	578 ± 5.79	0.341 ± 0.007	577 ± 5.21	0.512 ± 0.004	*P *> 0.05	*P *< 0.01

**Table 4:** Effect of surfactant type on the particle size of niosomes (Mean ± SD, n = 3).

**Formulation**	**Surfactant type**	**Size**
T40	Tween 60	646 ± 4.54
S40	Span 40	426 ± 2.18***
B40	Brij 72	466 ± 7.32***

* *P *< 0.05,

** *P *< 0.01 and

*** *P *< 0.001 compared to T40 formulation.

**Table 5:** Particle size of niosomes after storage at 4 °C and 25 °C (Mean ± SD, n = 3)

**Formulation**	**Storage**	**Size**			
		**0 day**	**7 day**	**21 day**	**28 day**
T40	4 °C	97 ± 7.43	98 ± 6.32	101 ± 8.32	104 ± 5.21
	25 °C		111 ± 5.29	116 ± 8.91	145 ± 7.32**
S40	4 °C	127 ± 5.87	129 ± 9.12	128 ± 8.01	134 ± 9.81
	25 °C		132 ± 9.43	138 ± 8.88	178 ± 9.12**
B40	4°C	161 ± 9.32	162 ± 8.21	466 ± 9.12***	569 ± 9.12***
	25 °C		168 ± 8.21	492 ± 9.23***	598 ± 7.21***

* *P* < 0.05,

** *P* < 0.01 and

*** *P* < 0.001 compared to 0 day.

**Table 6:** PDI of niosomes after storage at 4 °C and 25 °C (Mean ± SD, n = 3)

**Formulation**	**Storage**	**PDI**			
		**0 day**		**21 day**	**28 day**
T40	4 °C	0.220 ± 0.008	0.221 ± 0.006	0.211 ± 0.007	0.238 ± 0.005
	25 °C		0.231 ± 0.004	0.232 ± 0.008	0.301 ± 0.009*
S40	4 °C	0.265 ± 0.006	0.276 ± 0.004	0.281 ± 0.005	0.291 ± 0.007
	25 °C		0.287 ± 0.007	0.291 ± 0.008	0.498 ± 0.009**
B40	4 C	0.165 ± 0.006	0.198 ± 0.005	0.276 ± 0.004*	0.498 ± 0.005**
	25 °C		0.201 ± 0.006	0.431 ± 0.007**	0.598 ± 0.009***

* *P* < 0.05,

** *P* < 0.01 and

*** *P* < 0.001 compared to 0 day.

**Figure 2:**
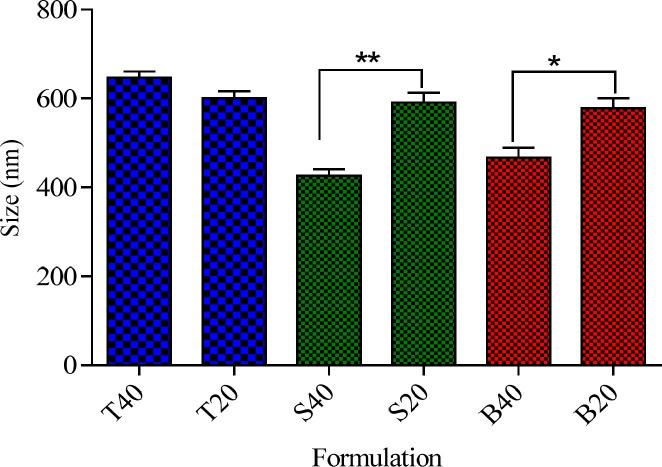
Effect of chol percentage on the particle size of the niosomes *P < 0.05, **P < 0.01 and ***P < 0.001 (Mean ± SD, n = 3)

**Figure 3:**
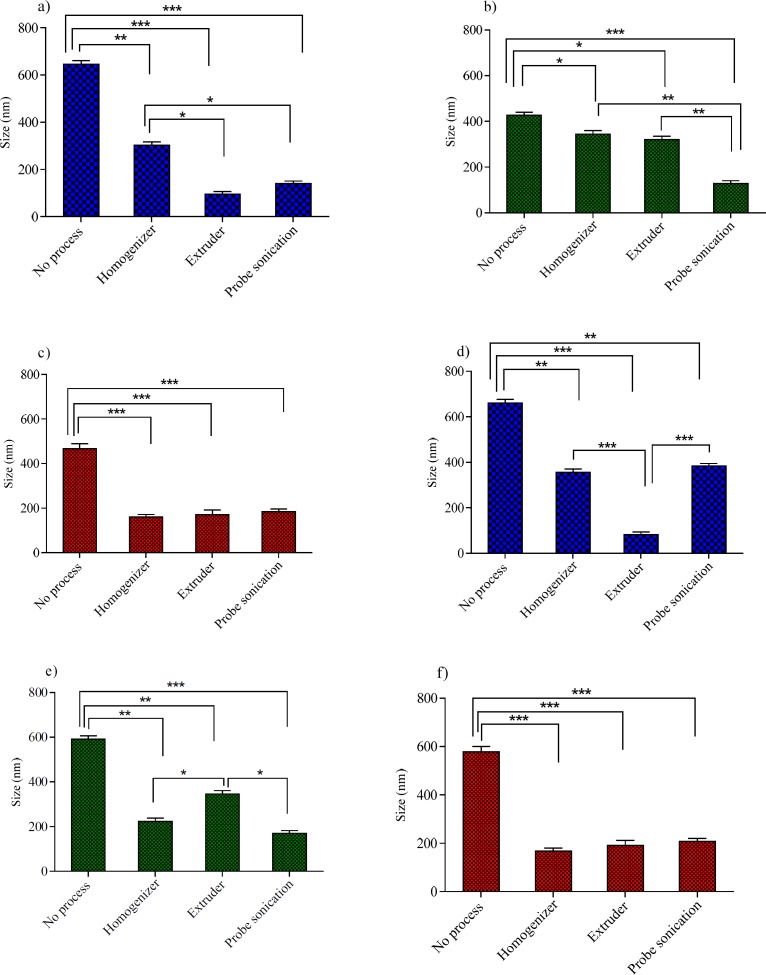
The influence of various size reduction methods on the particle size of niosomes with 40% Chol: (a) Tween 60 niosomes,(b) Span 60 niosomes and (c) Brij 72 niosomes and niosomes with 20% Chol: (d) Tween 60 niosomes, (e) Span 60 niosomes and (f) Brij 72 niosomes. ^*^P < 0.05, ^**^P < 0.01 and ^***^P < 0.001 (Mean ± SD, n = 3)

**Figure 4:**
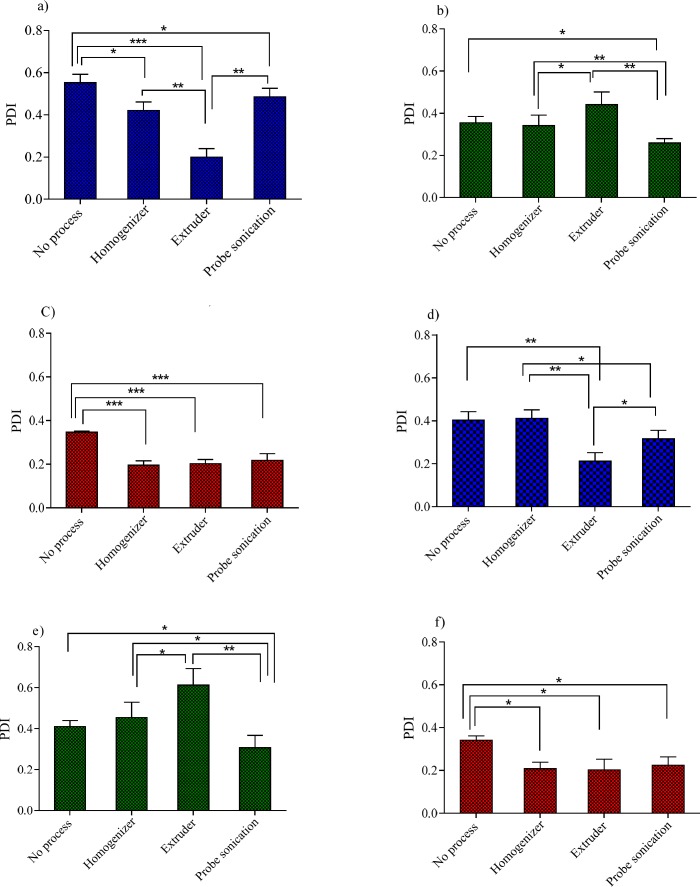
The influence of various size reduction methods on the PDI of niosomes with 40% Chol: (a) Tween 60 niosomes, (b) Span 60 niosomes and (c) Brij 72 niosomes and niosomes with 20% Chol: (d) Tween 60 niosomes, (e) Span 60 niosomes and (f) Brij 72 niosomes. ^*^P < 0.05, ^**^P < 0.01 and ^***^P < 0.001 (Mean ± SD, n = 3)

*Stability of niosomes*

The stability of niosomes (T40, S40 and B40) in terms of particle size and PDI was assessed. For this purpose, formulations were stored at temperatures of 4 °C and 25 °C for 28 day. Particle size and PDI of the samples were analyzed after 7, 21, and 28 day storage as described in the previous 

section. 

*Statistical analysis of data*

Data analysis was carried out using SPSS 17.0 software. Results were expressed as mean ± SD. Statistically significant difference was determined using the student t-test or one way ANOVA, *P *< 0.05 was set as a minimal level of significance.

## Results and Discussion

To investigate the effect of surfactant type, cholesterols content, and nanosizing method on the particle size and PDI of niosomes, various formulations were prepared using thin film hydration method. Composition of the prepared niosomes was shown in Table 2.

For each formulation the hydration process was carried out in either the presence or absence of bath sonication treatment and the effect of hydration method on size and PDI of the obtained niosomes were reported in Table 3. The results indicated that employing bath sonication during hydration process did not appear to have any significant effect on the vesicle size, while it had a significant effect on decrease in PDI for all niosomal formulations. The decrease in the PDI values indicates that the homogeneity of niosome size increases with applying bath sonication during the hydration process. These data emphasize the usefulness of bath sonication during the hydration process to obtain niosomes with a low PDI. In agreement with our findings, Lachataignerais *et al.* reported similar results about decrease of PDI with employing bath sonication (21). Therefore, bath sonication was applied during thin film hydration process in all further experiments.

*Effect of surfactant type on particle size of niosomes*

The effect of surfactant type on the particle size and PDI was presented in Table 4. As shown the type of surfactant significantly influenced the particle size of the niosomes. Based on the results, the size of niosomes showed a regular increase with an increase of the surfactant HLB values. Among the nonionic surfactants employed in the present study, Tween 60 has the highest HLB value of 14.9 and contains a lower hydrocarbon chain volume in comparison with the hydrophilic surface area while the relevant values for both Span 60 and Brij 72 are much lower, namely 4.7 and 4.9, respectively (Table 1). As it is clear from Table 4, the particle size of Tween 60 niosomes was about 48%, and 40% larger than those of Span 60 and Brij 72 niosomes (*P* < 0.05), indicating that at the same cholesterol content, niosomes composed of surfactants with a lower HLB value are expected to have smaller particle size than those with higher HLB values (22). 

*Effect of cholesterol percentage on the particle size of niosomes*

Cholesterol is one of the main components of niosomes that can influence their physicochemical characteristics and stability (23). Particle size is an important characteristic of vesicles from the pharmaceutical viewpoint. To study the effect of cholesterol content on the size of niosomes, a series of formulations containing two cholesterol percentages of 20% and 40% were prepared and the results were presented in Figure 2. As it can be observed irrespective of surfactant type, cholesterol was found to have significant effect on the particle size of the niosomes. However, the influence of cholesterol percentage on the size of niosomes was markedly dependent on the type of nonionic surfactant. For Tween 60 niosomes, an increase in the cholesterol percentage from 20 to 40% did not have any significant effect on the particle size (*P* > 0.05), while for the Brij 72 and Span 60 niosomes, increasing the amount of cholesterol caused a significant decrease in the average diameter of the particles. As shown in Figure 2, increasing cholesterol amount from 20 to 40 % caused about 20 and 27% decrease in the vesicle size of Brij 72 and Span 60 niosomes (591 ± 5.08 *vs.* 426 ± 2.18 and 578 ± 5.79 *vs.* 466 ± 7.32, respectively). This observation may be justified by the fact that the addition of cholesterol can enhance the bilayer hydrophobicity, leading to a decrease in the surface free energy and therefore decrease of particle size (22, 24). As regards for the Tween based formulations, it seems that because of the high hydrophilicity (high HLB), of this nonionic surfactant, the increase in the percentage of cholesterol was not enough to affect the hydrophobicity of bilayer, and therefore, no significant changes were observed in particle size of the relevant vesicles.

*The effect of size reducing methods on the particle size of niosomes*

In the process of niosomes preparation, often a size reducing method must be incorporated into the production procedure. A reduction in the vesicle size may be achieved by a number of methods; however, niosome composition is expected to play a critical role in the ability of the desired downsizing method. These considerations promoted us to examine the effects of three downsizing methods (probe sonicator, extruder and high pressure homogenizer) on the vesicle size and PDI of niosomes with various compositions. The obtained results for the particle size and PDI were shown in Figures 3 and 4, respectively.

For Tween 60 based formulations (Figures 3a, 3d, 4a and 4d), all the employed downsizing methods had significant effect on decreasing the size and PDI of the vesicles when compared with the untreated niosomes (*P* < 0.05). However, extrusion was found to be the most efficient method for reducing size and PDI of the Tween 60 niosomes. The nanoparticles obtained were smaller than 100 nm in size and showed obviously lower PDI values (Figures 4a and 4d), indicating the homogeneity of the vesicles (Z-average: 95 ± 2.56 nm, PDI: 0.189 ± 0.001 for T40 and Z-average: 82 ± 5.23 nm, PDI: 0.212 ± 0.004 for T20). Extrusion is used as the common approach for downsizing of liposomes (18). It has been reported that the gel-fluid transition temperature (T_c_) of the phospholipid composition along with the adjusted process temperature have impact on the efficiency of extrusion method (25). At lower temperatures than the T_c_, the rate of extrusion is usually slow, but at higher temperatures the rate is higher. The inability of extrusion below the phase transition temperature can be related to the much higher viscosity of gel-state membranes and their decreased deformability (26). The extrusion temperatures employed in the present study were all above the gel-fluid transition temperatures of the surfactants used.

The higher performance of extrusion for size reducing of T40 and T20 niosomes could be attributed to the lower T_c _value of Tween 60, which leads to formation of relatively fluid vesicles and consequently easier passage of vesicles through the polycarbonate filters of extruder than other formulations. The lack of efficiency of extrusion in the particle size reduction of Span 60 based formulation (S40 and S20) confirms the aforementioned suggestion further. Span 60 is solid at room temperature and has the highest T_c_ value (53 °C, Table 1) among the surfactants used in this study. As regards Span 60 niosomes, irrespective of Chol percentage, treating the vesicles with a probe sonicator led to better results (Z-average: 178 ± 5.49 nm, PDI: 0.261 ± 0.004 for S40 and Z-average: 170 ± 8.21 nm, PDI: 0.307 ± 0.005 for S20) (*P* < 0.05; compared with no process) (Figures 3b, 3e, 4b and 4e). 

For B40 and B20 formulations (Brij 72 based niosomes), as shown in Figures 3c, 3f, 4c, and 4f, the performance of all mentioned size reduction methods was almost similar and resulted in the same particle size and PDI values (about 200 nm and 0.2, respectively). The linear structure of Brij 72 and its intermediate T_c_ value may justify the observed data (Table 1).

*Stability testing*

Niosomes composed of 40% Chol, including T40, S40, and B40, which were downsized using extrusion, probe sonication, and high pressure homogenization, respectively were undertaken for stability studies. The reason for selection of formulations with a higher Chol content was that it can increase the microviscosity of the membrane by abolishing the gel-to-liquid phase transition of the surfactant bilayer, and therefore, results in a more stable and hydrophobic bilayer (27). For this purpose, niosomes were stored at temperatures of 4 °C and 25 °C for 28 day and particle size and PDI of the samples were analyzed after 7, 21 and 28 day storage. As shown in Tables 5 and 6, all formulations, except the Brij based niosomes, were stable at 4 °C over a 28 day period of time. While when stored at 25 °C, the size of all tested niosomes was increased significantly over a 21 day period. Increase in size may be related to the fusion and aggregation of vesicles during storage time. The current results showed that T40 and S40 were stable when maintained at 4 °C for at least 28 day. However, B40 formulation was unstable and became very turbid after 21 day incubation at both temperatures of 4 °C and 25 °C, more likely due to aggregation and fusion of the vesicles. 

The exact reasons for instability of the Brij based niosomes are not clear and further investigations are required to clarify the issue more. 

## Conclusion

Surfactant composition, cholesterol content, and the type of particle size reduction technique had significant effects on the particle size and PDI of the prepared niosomes.

With an increase in the surfactant HLB values, a regular increase in particle size of niosomes was shown. At the same cholesterol content, niosomes composed of Span 60 and Brij 72 with lower HLB values (about 4.8) showed markedly smaller particles size when compared with those composed of Tween 60 (HLB = 14.9). As regards the influence of cholesterol content and the employed downsizing method, the results showed that the influence of both parameters on the size of niosomes was markedly dependent on the type and more likely on the HLB and T_c _values of nonionic surfactant. For Tween 60 niosomes as a hydrophilic surfactant, an increase in the cholesterol percentage did not have any significant effect on the particle size (*P* > 0.05), while for the Brij 72 and Span 60 niosomes, increasing the amount of cholesterol caused a significant decrease in the average diameter of the particles. To obtain niosomes with a small particle size and narrow PDI, the extrusion was more appropriate technique for those vesicles composed of Tween 60 which is a flexible and relatively fluid surfactant (T_c_ = 21 °C), while probe sonication was more desirable for niosomes composed of Span 60 which is a relatively rigid surfactant (T_c _= 53 °C). 
